# Effects of California’s New Patient Homelessness Screening and Discharge Care Law in an Emergency Department

**DOI:** 10.7759/cureus.35534

**Published:** 2023-02-27

**Authors:** McKenzie Eakin, Vania Singleterry, Ewen Wang, Ian Brown, Olga Saynina, Rebecca Walker

**Affiliations:** 1 Emergency Medicine, Emory University School of Medicine, Atlanta, USA; 2 Pediatric Emergency Medicine, University of California San Francisco, Oakland, USA; 3 Emergency Medicine, Stanford University School of Medicine, Palo Alto, USA; 4 Health Policy, The Stanford Center for Policy, Outcomes and Prevention, Palo Alto, USA

**Keywords:** screening program, social determinants of health (sdoh), public health and social work, health-care policy, common emergency department complaints, person experiencing homelessness (peh)

## Abstract

Introduction

California State Bill 1152 (SB1152) mandated all non-state-operated hospitals meet specific criteria when discharging patients identified as experiencing homelessness. Little is known about SB1152’s effect on hospitals or compliance statewide. We studied the implementation of SB1152 in our emergency department (ED).

Methods

We analyzed our suburban academic ED’s institutional electronic medical record for one year before (July 1, 2018-June 20, 2019) and one year after (July 1, 2019-June 30, 2020) implementation of SB1152. We identified individuals by lack of address during registration, International Classification of Diseases, Tenth Revision (ICD-10) code of homelessness, and/or the presence of an SB1152 discharge checklist. Demographics, clinical information, and repeat visit data were collected.

Results

ED volumes were constant during the pre- and post-SB1152 periods (approximately 75,000 annually); however, ED visits by people experiencing homelessness more than doubled (630 (0.8%) to 1530 (2.1%) in the pre- and post-implementation periods. Age and sex distributions were similar with approximately 80% of patients aged 31-65 years and less than 1% under 18. Visits by females comprised less than 30% of the population. Visits by people of the White race decreased from 50% to 40% pre- and post-SB1152. Visits by people of the Black, Asian, and Hispanic races experiencing homelessness increased by 18% to 25%, 1% to 4%, and 19% to 21%, respectively. Acuity was unchanged with 50% of visits classified as “urgent.” Discharges increased from 73% to 81% and admissions halved from 18% to 9%. Visits by patients with only one ED visit decreased (28% to 22%); those with four or more visits increased (46% to 56%). The most common primary diagnoses pre- and post-SB1162 were alcohol use (6.8% and 9.3%, respectively), chest pain (3.3% and 4.5%, respectively), convulsions (3.0%, and 2.46%, respectively), and limb pain (2.3% and 2.3%, respectively). The primary diagnosis of suicidal ideation doubled from the pre- to post-implementation periods (1.3% to 2.2%, respectively). Checklists were completed for 92% of identified patients discharged from the ED.

Conclusion

Implementation of SB1152 in our ED resulted in identifying an increased number of persons experiencing homelessness. We identified opportunities for further improvement since pediatric patients were missed. Further analysis is warranted, especially with the coronavirus disease 2019 (COVID-19) pandemic, which has significantly affected healthcare-seeking behavior in EDs.

## Introduction

Over half a million people in the United States (US) experience homelessness on any given day, with a 28% increase in specifically unsheltered individuals in the last five years [1]. Over one-quarter live in California [1]. The increase of unsheltered individuals has coincided with a renewed nationwide concern for the practice of "patient dumping," where patients may be sent home from an emergency department (ED) or hospital visit without means for safe, sheltered healing [2]. California Senate Bill 1152 (SB1152) mandated all non-state-operated hospitals meet and document specific criteria on a "discharge checklist" when discharging patients identified as experiencing homelessness [3].

Little is known about the demographic or medical characteristics of patients experiencing homelessness seen in our academic, suburban ED in northern California. Additionally, SB1152 does not specify how an individual hospital should quantify compliance. We sought to describe this patient population and compliance with SB1152 in our ED. 

## Materials and methods

The study was conducted at Stanford University, Palo Alto, California, United States. The principal investigator (RW) queried all stakeholders (registration, nurses, social workers, and information technology staff/EPIC superuser (Epic Systems Corporation, Verona, Wisconsin, United States)) regarding how homelessness was assessed in our ED and hospital system. The information workflow for all persons experiencing homelessness was determined. To identify the cohort of patients experiencing homelessness, we used several methods. We searched our ED discharge record for “homeless” or a value of <empty> in the address field, and also searched for a "dummy" zip code that the department uses to signify that no home zip code was entered during registration. We also included those with an International Classification of Diseases, Tenth Revision (ICD-10) diagnosis code of homelessness. After July 1, 2019, we queried the electronic medical record (EMR) media section for the presence of a homeless discharge checklist within an encounter. This project was part of a quality improvement project and was determined exempt from IRB oversight for Human Subject Research.

We analyzed our institutional EMR one year pre- and post-SB1152 implementation. This time period included July 1, 2018-June 30, 2019 for the year prior to the law going into effect and the period of July 1, 2019-June 30, 2020, for the year after implementation. After identifying the encounters through the above method, we collected and compared data in the EMR regarding patient demographic categories of age (<18, 18-65, and >65 years), sex (female, male, other), self-reported race/ethnicity (White, Black, Asian, Hispanic, Other), preferred language (English, Spanish, Other), and primary insurance (Medicaid, Medicare, Uninsured, Private, Other). We also analyzed ED utilization (number of visits per patient for the study year, i.e., 1, 2, 3, 4+ visits), and clinical information. Clinical data included the assigned triage Emergency Severity Index (ESI) score (1-Resuscitation; 2-Emergent, 3-Urgent, 4-Semi-Urgent, 5-Non-Urgent, missing) and primary diagnosis (International Classification of Diseases, Ninth Revision (ICD-9), and disposition (Discharged, Admitted, Other) data for all patients identified as experiencing homelessness.

Analyses

Chi-square tests were performed to compare the Pre- and Post-SB1152 populations according to demographic, utilization, and clinical factors [4]. In a subanalysis, we compared the primary diagnosis (ICD-9) of all patients compared to those with 4+ visits. We also tabulated checklist completion rates by patient disposition and location of checklist completion (ED vs inpatient).

## Results

Our suburban, academic ED had a yearly volume of approximately 76,000 visits and 52,000 unique patients during both periods. The number of patients experiencing homelessness identified in the ED more than doubled pre- and post-SB1152 from 290 to 691 (0.5% to 1.3% of all patients, respectively). Unique patient visits increased 143% pre- and post-SB1152 from 630 to 1530 (Table 1).

**Table 1:** Unique visits by patients experiencing homelessness during the study period, July 2018 through June 2020, pre- and post-SB1152 (Total n=2160)

Unique Visits	Pre-implementation: July 2018- June 2019		Post-implementation: July 2019-June 2020	
	n	%	n	%
Total	630	100%	1530	100%
Age (years)				
>65	76	12%	157	10%
18-65	552	88%	1365	89%
<18	2	0%	8	1%
Gender				
Female	177	28%	432	28%
Male	452	72%	1098	72%
Other	1	0%	0	0%
Race/Ethnicity				
Non-Hispanic White	315	50%	617	40%
Non-Hispanic Black	115	18%	386	25%
Non-Hispanic Asian	9	1%	60	4%
Hispanic	122	19%	327	21%
Other/Unknown	69	11%	140	9%
Language				
English	530	84%	1376	90%
Spanish	88	14%	141	9%
Other language	12	2%	13	1%
Primary Insurance				
Medicaid	448	71%	1080	71%
Medicare	111	18%	296	19%
Uninsured	54	9%	121	8%
Private & Other	17	3%	33	2%
Triage Emergency Severity Index				
1-Resuscitation	7	1%	10	1%
2-Emergent	125	20%	310	20%
3-Urgent	333	53%	785	51%
4-Semi-Urgent	123	20%	333	22%
5-Non-Urgent	34	5%	66	4%
ESI Missing	8	1%	26	2%
Disposition				
Discharged	445	71%	1239	81%
Admitted	111	18%	140	9%
Other	74	12%	151	10%
Number of Visits				
1 visit	176	28%	342	22%
2-3 visits	164	26%	331	22%
4+ visits	290	46%	857	56%

In pre- and post-SB1152 periods, age and sex distributions were similar with approximately 80% of patients aged 31-65 years and less than 1% of patients under 18. Visits by females comprised less than 30% of the population. Visits by persons of the White race decreased from 50% to 40%, while visits by the Black, Asian, and Hispanic races increased from 18% to 25%, 1% to 4%, and 19% to 21%, respectively. Initial triage acuity did not change with 50% of visits designated Urgent and 21% Emergent or Critical, for both periods. Discharges increased from 73% to 81% and admissions halved from 18% to 9%. Patients with only one ED visit decreased (28% to 22%) while those with four or more visits increased by 10% (46% to 56%). 

The most common primary diagnoses for the study population during both study periods were alcohol use (6.8% and 9.3%, respectively), chest pain (3.3% and 4.5%, respectively), convulsions (3.0% and 2.46%, respectively), and limb pain (2.3% and 2.3%, respectively) (Table 2). The primary diagnosis of suicidal ideation nearly doubled from the pre- to post-implementation periods (1.3% to 2.2%, respectively). Although the most frequent visitors’ most common diagnoses were largely similar, the post-SB1152 period saw increases in the proportion of this population presenting with alcohol use, chest pain, and suicidal ideation.

**Table 2:** Most common primary diagnoses of visits by all patients experiencing homelessness and those with 4+ visits (Total n=2160)

Diagnoses	July 2018-June 2019		July 2019-June 2020	
	All	4+ Visits	All	4+ Visits
Alcohol use	6.8%	6.9%	9.3%	10.5%
Chest pain	3.3%	3.4%	4.5%	6.2%
Convulsions	3.0%	5.5%	2.5%	3.9%
Pain in limb	2.2%	3.1%	2.2%	2.1%
Lack of housing	2.2%	2.8%	2.0%	1.8%
Suicidal ideation	1.3%	0.3%	2.2%	2.0%

Checklists were completed for approximately 92% of visits of patients discharged from the ED.

## Discussion

To our knowledge, California SB1152 is the first state to have mandated minimum standards for screening and care of people experiencing homelessness upon hospital discharge. Upon implementation of SB1152, our institution increased its identification of patients experiencing homelessness two-fold. While the demographics of our study population generally followed the profile of persons experiencing homelessness in our county, we identified significant gaps, such as no pediatric patients during both periods. Clinically, we found no change in visit acuity and a significant decrease in admission rate. Overall compliance of our ED with the state SB1152 mandate was high.

The increase in identified ED visits by this population is most likely due to an increase in the identification of persons experiencing homelessness through the new policies and procedures. We believe that in-services regarding SB1152 and the need to identify patients and fill out the checklist resulted in an increased institutional commitment to identify these vulnerable patients. Other contributing factors could include an overall increase in persons experiencing homelessness over the study period; however, our specific county rates have been stable in recent years [5-7]. An increase in ED visits by a stable population is also unlikely given that unique visits and unique patients increased comparably. Furthermore, although the implementation of SB1152 coincided with the first three months of the coronavirus disease 2019 (COVID-19) pandemic, which could have caused a decrease in access to ambulatory health services and increased ED visits, the rate of visits for the last three months of the study period was not markedly increased.

The rate of ED visits by persons experiencing homelessness is not well characterized. There are few studies that document anywhere from 7-9% in a suburban and 18% in a central city ED in the Northeast, and 25% in a Southeast safety net hospital [8,9]. Our study is in a suburban setting in California where scant data has been collected with regard to patients experiencing homelessness. Our ED draws patients from two surrounding counties with an estimated prevalence of people experiencing homelessness of 0.2% and 0.5%, respectively [5-7]. Although we would expect an overrepresentation of this vulnerable population in any healthcare setting including the ED, we may be serving relatively fewer of these patients within our county due to nearby county safety-net hospitals. Nonetheless, the epidemiology of our identified population is generally analogous to that identified by county studies.

Our institution, however, probably still undercounts the true number of persons experiencing homelessness, partly due to different definitions of “homeless.” Much of the relevant research base is based on the National Hospital Ambulatory Medical Care Survey (NHAMCS) definition (“no home (e.g., lives on the street) or (the) patient's current place of residence is a homeless shelter”) and data [9]. Although there is significant movement toward a broader definition of housing instability both in research and in policy (including but not limited to inclusion of people who sleep in cars, or unhealthily crowded sheltered living situations, i.e., two or more families in a single-family home, known as “doubling up”), SB1152 uses more narrow language [3,10]. Furthermore, patients are primarily identified at registration in our ED, which effectively serves as a screening process that is universal but not standardized. Thus, patients in need might not be identified or offered assistance.

There are several possible reasons for our lack of identified pediatric patients in this study. First, families might be housing unstable, sleeping in cars, or doubling up, but not identified because they have some viable address to give. Children and youth experiencing homelessness are well known to avoid self-disclosure and may avoid medical care for trust and safety concerns. The best-case screening scenarios are those protocolized with language specific to this population, performed by trained screeners [11,12]. Further study is needed to better understand how to most accurately identify and assist this special subpopulation.

Patients experiencing homelessness had similar diagnoses pre- and post-SB1152. Frequent users, defined as those with four or more ED visits per year, accounted for 56% of the visits post SB1152. As this is the first known statewide mandate of a minimum standard of screening and care, future studies may elucidate whether implementation of the protocol reduces revisit rates, whether different hospitals are affected equally, and whether there are better health outcomes or reduced costs over a longer timeframe. A more granular study of the checklist data may illuminate which items are most “in demand,” informing and expediting future care delivery [13-15].

There is no language in SB1152 specifying checklist completion metrics or requirements to be considered compliant. While we are encouraged by our progress and compliance thus far, we recognize opportunities for improvement. Improving compliance across all discharges is important not just legally, but also, to understand how compliance correlates with improved health outcomes like reduced ED visits. This is particularly important for identifying children and families with children since homelessness directly and deeply affects school and physical and mental health.

Limitations

This study is subject to limitations. This is a quality assessment of one site but gives insight into operationalizing screening, including its benefits and our opportunities for improvement. As evidenced by the near-absence of identified pediatric patients, we are still not capturing the entirety of the relevant population. Second, the COVID-19 pandemic may have affected visit volumes and diagnoses for the ED overall and specific to this population in the latter part of the post-SB1152 period. The effect of the efforts surrounding the new protocol on cost and length of stay was not analyzed in this study, but may be important.

## Conclusions

We provide an assessment of our institution’s implementation of SB1152. Our institution doubled identification of patients experiencing homelessness overall, but failed to identify children. The ED had high compliance with checklist completion. Going forward, understanding what elements of the checklist are most commonly utilized may provide insight into how we can better care for this population. 

## References

[1] Henry M, de Sousa T, Roddey C, Gayen S, Bednar TJ (2022). Homeless in California Statistics 2020. Homeless Estimation by State | US Interagency Council on Homelessness. Accessed. The 2020 Annual Homeless Assessment Report (AHAR) to Congress. Part 1: Point-in-time Estimates of Homelessness.

[2] Moulin A, Lopez-Gusman E (2019). How a bill becomes a law, or how a truly terrible bill becomes less awful. West J Emerg Med.

[3] (2022). SB-1152 Hospital Patient Discharge Process: Homeless Patients. https://leginfo.legislature.ca.gov/faces/billTextClient.xhtml?bill_id=201720180SB1152.

[4] (2022). Chi-Square Calculator. https://www.socscistatistics.com/tests/chisquare/default2.aspx.

[5] (2022). 2019 One Day Homeless Count. https://hsa.smcgov.org/2019-one-day-homeless-count.

[6] (2022). Santa Clara County Homeless Census and Survey Reports: Homeless census and survey. https://osh.sccgov.org/continuum-care/reports-and-publications/santa-clara-county-homeless-census-and-survey-reports.

[7] (2022). U.S. Census Bureau QuickFacts: San Mateo County, California; Santa Clara County, California. https://www.census.gov/quickfacts/fact/table/sanmateocountycalifornia,santaclaracountycalifornia/PST045222.

[8] Feldman BJ, Calogero CG, Elsayed KS (2017). Prevalence of homelessness in the emergency department setting. West J Emerg Med.

[9] Jackson TS, Moran TP, Lin J, Ackerman J, Salhi BA (2019). Homelessness among patients in a southeastern safety net emergency department. South Med J.

[10] (2022). California Code, Health and Safety Code - HSC § 1262.4. https://codes.findlaw.com/ca/health-and-safety-code/hsc-sect-1262-4.html.

[11] Beharry MS, Christensen R (2020). Homelessness in pediatric populations: strategies for prevention, assistance, and advocacy. Pediatr Clin North Am.

[12] Nam E, Palmer AN, Patel M (2021). Characteristics of emergency department visits by homeless young adults in the U.S. J Adolesc Health.

[13] Franco A, Meldrum J, Ngaruiya C (2021). Identifying homeless population needs in the emergency department using community-based participatory research. BMC Health Serv Res.

[14] Miyawaki A, Burke LG, Khullar D, Tsugawa Y (2020). Comparison of 30-day readmission and emergency department revisit rates among homeless patients at teaching versus non-teaching hospitals. Soc Sci Med.

[15] Salhi BA, White MH, Pitts SR, Wright DW (2018). Homelessness and emergency medicine: a review of the literature. Acad Emerg Med.

